# Induction techniques that reduce redistribution hypothermia: a prospective, randomized, controlled, single blind effectiveness study

**DOI:** 10.1186/s12871-019-0866-8

**Published:** 2019-11-06

**Authors:** Jonathan V. Roth, Leonard E. Braitman, Lacy H. Hunt

**Affiliations:** 1 Department of Anesthesiology, Albert Einstein Medical Center, 5501 Old York Road, Philadelphia, PA USA; 2 Albert Einstein Healthcare Network, Philadelphia, PA USA; 3 Sidney Kimmel Medical School of Thomas Jefferson University, Philadelphia, PA USA; 4 Office for Research and Technology Development, Albert Einstein Medical Center, 5501 Old York Road, Philadelphia, PA USA

**Keywords:** Hypothermia, Redistribution hypothermia, Intraoperative hypothermia, Perioperative hypothermia, Postoperative hypothermia, Anesthesia induction, Inhalation induction, Intravenous induction, Inhalation anesthesia induction, Intravenous anesthesia induction

## Abstract

**Background:**

While much effort has been devoted to correcting intraoperative hypothermia, less attention has been directed to preventing redistribution hypothermia. In this study, we compared three different anesthetic induction techniques to standard IV propofol inductions (control) in their effect on reducing redistribution hypothermia.

**Methods:**

Elective, afebrile patients, age 18 to 55 years, were randomly assigned to one of four groups (*n* = 50 each). Group “INH/100” was induced with 8% sevoflurane in 100% oxygen, Group “INH/50” with 8% sevoflurane in 50% oxygen and 50% nitrous oxide, Group “PROP” with 2.2 mg/kg propofol, and Group “Phnl/PROP” with 2.2 mg/kg propofol immediately preceded by 160 mcg phenylephrine. Patients were maintained with sevoflurane in 50% nitrous oxide and 50% oxygen in addition to opioid narcotic. Forced air warming was used. Core temperatures were recorded every 15 min after induction for 1 h.

**Results:**

Compared to control group PROP, the mean temperatures in groups INH/100, INH/50, and Phnl/PROP were higher 15, 30, 45 and 60 min after induction (*p* < 0.001 for all comparisons), averaging between 0.39 °C and 0.54 °C higher. In group PROP, 60% of patients had at least one temperature below 36.0 °C in the first hour whereas only 16% did in each of groups INH/100, INH/50, and Phnl/PROP (*p* < 0.0001 in each group compared to PROP).

**Conclusions:**

In this effectiveness trial, inhalation inductions with sevoflurane or with prophylactic phenylephrine bolus prior to propofol induction reduced the magnitude of redistribution hypothermia by an average of 0.4 to 0.5 °C in patients aged 18 to 55 years.

**Trial registration:**

Retrospectively registered on clinical-trials.gov as NCT02331108, November 20, 2014.

## Prior presentations

1. Roth JV, Braitman LE: Induction techniques that can reduce redistribution hypothermia. Abstract presented at the American Society of Anesthesiologists Annual Meeting, October 23, 2016.
2. Roth JV, Braitman LE, Hunt LH: Induction techniques that can reduce redistribution hypothermia. Abstract presented at the International Anesthesia Research Society Annual Meeting, #1135, May 2017.

The Pennsylvania Society of Anesthesiologists has published a non-peer reviewed summary letter of this work in their newsletter based upon data from the abstract submissions. There is a very small amount of text recycling.

## What we already know about this topic

Propofol causes significant redistribution hypothermia, and intraoperative hypothermia is common in the first hour of anesthesia.

## What this article tells us that is new

Either inhalation inductions with sevoflurane or the administration of phenylephrine immediately prior to intravenous induction with propofol can reduce the degree of redistribution hypothermia by an average of 0.4 to 0.5 °C during the first hour of anesthesia. The degree and duration of intraoperative hypothermia can be reduced by using these alternative induction techniques.

## Background

Hypothermia has multiple adverse consequences and should be avoided [1, 2]. Anesthesia induction with propofol is known to cause a rapid and clinically important temperature decrease due to redistribution hypothermia, typically by about 1.5 °C [3]. Ikeda et al. showed there is on average 0.7 °C less redistribution hypothermia when patients are induced with an inhalation induction rather than with intravenous propofol [3]. However, the use of inhalation inductions has not been widely adapted. Sun et al. documented that hypothermia is routine during the first hour of anesthesia [4]. While there is great effort expended to warm patients intraoperatively, relatively little attention has been directed to preventing redistribution hypothermia.

In studies assessing whether patients were hypothermic, two methods have been used. First, the “end of case” temperature has been used to assess hypothermia and its association with complications [5]. In the United States, the SCIP (Surgical Care Improvement Project) guideline for body temperature management uses end of case temperature as its measure of compliance [5]. However, this method has limitations in assessing intraoperative hypothermia. Some hypothermia complications occur intra-operatively (e.g., coagulopathy, increased transfusion requirements), some post-operatively (e.g., shivering, delayed emergence) and some likely both (e.g., infection risk) [4, 6, 7]. End of case hypothermia indicates intraoperative hypothermia. End of case normothermia does not imply intraoperative normothermia. A patient may have been hypothermic intraoperatively, having suffered the consequences of intraoperative hypothermia, achieving normothermia only at the end of the case. Thus, the contribution of intraoperative hypothermia to postoperative complications may be unrecognized.

Because of the limitations of using end of case temperature as an indication of intraoperative hypothermia, more current literature focuses on the area under the core temperature vs time curve. The magnitude of the area under the time vs core temperature curve below a threshold, typically 36.0 °C, is used as an indicator of the degree of hypothermia. The greater the area under 36.0 °C, the greater the amount of intraoperative hypothermia. It is plausible that if redistribution hypothermia can be reduced, there will be less intraoperative hypothermia (assessed by less area under the curve) and thus fewer intraoperative and postoperative complications associated with hypothermia.

Vasodilation causes redistribution hypothermia by increasing blood flow to the cooler peripheral and dermal thermal compartments. This results in heat transfer away from the warmer core. We hypothesized that anesthetic inductions causing less vasodilation (than propofol alone inductions) will result in less redistribution hypothermia. The purpose of this effectiveness study is to compare the effect of three such alternative induction techniques to standard propofol inductions on core temperature during the first hour of anesthesia.

## Methods

This study and consent forms were approved by our IRB (Albert Einstein Healthcare Network IRB #1) and submitted to clinical-trials.gov as NCT02331108 by Jonathan V. Roth on November 20, 2014. Written informed consent was obtained by the first author from all participating patients. (The consents were all dated and timed and signed by the patient and the first author. A copy of the consent was provided to the patient. The original signed consent was maintained in the research file.) The manuscript complies with the CONSORT requirements. This study was performed at the Albert Einstein Healthcare Network in Philadelphia, Pennsylvania during 2014 and 2015.

Four groups (described below and summarized in Table 3 in Appendix 1) of 50 patients each were studied. The major inclusion criteria were: age 18 to 55 years inclusive; supine or lithotomy positioning; scheduled for general anesthesia where 50% nitrous oxide would be used; endotracheal intubation or laryngeal mask airway insertion would be used; afebrile (preoperative oral or temporal scan temperature between 36.2 and 37.4 °C inclusive); forced air warming would be used; and expected duration of anesthetic to be at least 60 min. A complete list of inclusion and exclusion criteria are presented in Table 4 in Appendix 2. After enrollment, random assignments were contained in opaque envelopes that were opened immediately before induction of anesthesia. Each of the envelopes contained one of the four group designations, 50 envelopes for each group. Randomization was achieved by putting the envelopes in a basket and mechanically mixing the envelopes within the basket. When a patient was entered into the study, an opaque envelope was selected arbitrarily from any location in the stack.

For all patients, operating rooms were kept between 21 °C and 24 °C with a target of 22 °C. No patients were prewarmed. Upon entering the operating room, cotton blankets were placed on all patients covering their lower extremities, abdomen, and thorax. These blankets were removed after induction to allow for forced air warming (FAW) blanket placement and surgical positioning, preparation, and draping. All operating rooms had the same air flow design. Patients were administered 2 mg IV midazolam prior to entering the operating room. No opioid narcotics were administered until after the airway was secured with either a laryngeal mask airway (LMA) or endotracheal tube. Heat and moisture exchangers were used on all patients. Patients could receive up to 300 mL room temperature intravenous crystalloid before fluid was warmed (Ranger, Arizant Healthcare, Eden Prairie, MN) to 41 °C. All inductions, nasal temperature probe placement, and application of a FAW blanket were performed in the same manner by the first author. (Nasal temperature was used as a surrogate for core temperature for all patients since it could be used for patients having either an LMA or endotracheal intubation [8].) Either an upper or lower body FAW blanket (SW-2010 Snuggle Warm Small Upper Body Convective Warming Blanket, or SW-2001 Snuggle Warm Adult Full Body Convective Warming Blanket, Level 1, Smiths Medical ASD, Rockland, MA) was used. The face was not directly covered by the FAW blanket in order to avoid the possibility that a collection of warm air could affect the nasal temperature measurements. Cotton blankets were placed on top of the warming blankets. The FAW (Equator Convective Warmer, Level 1, Smiths Medical ASD, Rockland, MA) was turned on to 44 °C as soon as the patient was prepped and draped; the time duration from the start of induction (T_0_) until the time the FAW was turned on was recorded. Neurophysiologic monitors to measure “depth of anesthesia” were not used. Pre-induction core temperatures were not measured. All doses of propofol were based on actual (not ideal) body weight.

### Group INH/100 – inhalation induction with sevoflurane in 100% oxygen (O_2_)

A baseline blood pressure was taken prior to induction. No formal preoxygenation regimen was performed. The patients were asked to breath for a few breaths via the face mask with 100% O_2_ just to confirm reservoir bag movement and capnograph detection of carbon dioxide. At time T_0_, with an unprimed circuit, the O_2_ flow meter was set at 6 LPM and the sevoflurane vaporizer was turned on at 8%. Blood pressures were recorded every minute starting 1 min after T_0_ (T_1_) until airway intervention commenced. At the discretion of the first author, an LMA was inserted when the patient was assessed to be adequately deep, determined by masseter muscle relaxation, typically just 2 min after T_0_ (T_2_). Alternatively, if the patient was to be endotracheally intubated, muscle relaxant (vecuronium, rocuronium, or succinylcholine) was administered when the patient was assessed as being unconscious, typically at T_1_. Positive pressure ventilation was performed as required until endotracheal intubation. If necessary, to avoid hypotension, the Sevoflurane concentration was decreased while waiting for adequate muscle relaxation. If the systolic blood pressure dropped below 85 mmHg prior to airway intervention, the patient would be treated immediately either with phenylephrine or airway intervention if ready. After securing either the LMA or endotracheal tube, anesthesia was maintained with sevoflurane in 50% nitrous oxide (1 LPM) and 50% O_2_ (1 LPM). Opioid narcotics (fentanyl, hydromorphone, methadone), neuromuscular reversal agents (glycopyrrolate, neostigmine), dexamethasone, and ketamine were administered as per the discretion of the attending anesthesiologist.

A skin temperature probe (Skin Temperature Sensor, 400 Series, DeRoyal Industries, Inc., Lane Powell, TN) was modified by removing the skin adhesive portion, bending the probe 90° 8 cm from the tip (to assure insertion depth would be 8 cm), and straightening the probe from the bend to the tip. Previous work has shown a close agreement between the nasal technique used in this study and distal esophageal temperature measurements [8]. Within 10 min of T_0_, the blunt tipped nasal temperature probe was inserted 8 cm into one naris [8–10]. This provided a minimum of 5 min for thermal equilibration of the temperature probe before the first measurement (T_15_), 15 min after T_0_. Either naris was used arbitrarily. Starting at T_15_, nasal temperatures were recorded every 15 min (T_15_, T_30_, T_45_, T_60_). If the core temperature reached 37.5 °C, the FAW was turned off. The patient’s data were included in the analysis if there were at least two temperature measurements (T_15_ and T_30_). If the anesthetic ended before 30 min or if there was a protocol violation, that patient’s data were not analyzed; a replacement envelope assigning another future patient to that group was generated and inserted randomly back into the envelope stack. All patients received 4 mg ondansetron within 15 min of emergence. Temperature data collection ceased at the initiation of IV acetaminophen administration or if there was any event that could have a substantial impact on patient temperature. All cystoscopy procedures were conducted with warmed bladder irrigation.

### Group INH/50 - inhalation induction with sevoflurane in 50% nitrous oxide (N_2_O) / 50% O_2_

The protocol was identical to group INH/100 except that induction was performed with 3 LPM N_2_O and 3 LPM O_2_ (instead of 6 LPM O_2_) with 8% sevoflurane.

### Group PROP – intravenous induction with 2.2 mg/kg intravenous propofol

The induction differed from group INH/100 in the following manner. Two mL of 2% lidocaine (40 mg) were added to 20 mL of 1% propofol. After preoxygenation with 100% O_2_ for a minimum of 2 min, 3 mL of 2% lidocaine (60 mg) was administered followed immediately by 2.2 mg/kg propofol (rounded to the nearest 5 mg) at T_0_. If the patient was to receive an LMA, one blood pressure was taken at T_1_ and then the LMA was inserted. If the patient was to be endotracheally intubated, muscle relaxant was administered immediately after propofol administration, blood pressures were measured every minute, and positive pressure ventilation with 100% O_2_ was performed as required. After securing the airway, the protocol continued in the same manner as in Group INH/100.

### Group Phnl/PROP – intravenous induction with 2.2 mg/kg intravenous propofol preceded by 160 mcg phenylephrine

The protocol differed from group PROP only in that 2 mL of 80 mcg/mL phenylephrine (160 mcg) was administered immediately after the administration of 3 mL 2% lidocaine but before the 2.2 mg/kg propofol.

## Statistical methods

To address the lack of pre-test core temperature measurements, we used the post-test only, single blind randomized trial. This is a “true experimental design” [11]. The primary outcomes were the nasal (core) temperatures at 4 time points after induction (not changes from pre-induction baseline). In bivariate analyses, we compared differences in mean core temperature between the propofol only induction control group (PROP) and each of 3 groups administered alternative induction techniques (INH/100, INH/50, and Phnl/PROP). Specifically, analyses of the mean temperature differences (and 95%CIs) for 1) INH/100 vs. PROP, 2) INH/50 vs. PROP, and 3) Phnl/PROP vs. PROP were performed at each of 15, 30, 45, and 60 min (T_15_, T_30_, T_45_, and T_60_) after induction. These differences in mean core temperatures at T_15_, T_30_, T_45_, and T_60_ among groups were assessed using unpaired t-tests and corresponding 95% confidence intervals (95% CIs). Bonferroni’s correction was used to adjust for these 12 multiple comparisons. Core temperature data were tested for normality by the Shapiro-Wilk test.

The random assignment of 50 patients per group made it likely that the treatment groups would be balanced in both measured and unmeasured characteristics (including pre-induction core temperatures). However, imbalances did occur in BMI and sex. Those imbalances and the lack of pre-induction core temperature measurements necessitated a multivariable analysis comparing the average core temperatures at T_15_, T_30_, T_45_, and T_60_; the covariates were BMI, sex, age, ASA classification, and time to initiating FAW. (Upper vs lower FAW were not covariates because the rates of heat transfers are similar [12].) This multivariable analysis was a linear mixed model with random intercepts and random slopes and unstructured covariance. This model fit better than a model with random intercepts alone nested within it (*p* < 0.0001 by the likelihood ratio test) [13]. There was no statistically significant interaction between group and time (*p* = 0.15). Since the results of the bivariate and multivariable analyses were similar and led to the same conclusions, we present the simpler bivariate results. The differences in the secondary outcomes, the percentages of patients who had at least one temperature < 36.0 °C (and ≤ 35.5 °C) between the control group (PROP) and each of the other three groups were evaluated by Fisher’s exact tests. Although the resulting *p* values were exact; the corresponding 95% CIs were approximate.

Interval estimates of the percentages of patients that developed hypotension requiring treatment and of patients undergoing an inhalation induction who developed apnea were computed using exact binomial 95% CIs.

In a statistical power analysis, assuming alpha = 0.05 and beta = 0.2, 17 patients per group were needed to detect a 0.5 °C difference between means of any two compared groups as statistically significant in a two tailed test. We enrolled patients who were expected to have a surgical procedure lasting at least 60 min after the induction of anesthesia. However, we had no way to estimate the percentage of cases that would end early and therefore not provide data at T45 and T60. We increased the size of the study groups to 50 in order to be reasonably sure there were at least 17 patients in all groups at T60.

All statistical analyses compared patients as treated using two-sided tests with alpha = 0.05 and were performed using Stata, version 14 (Stata Press, College Station, Texas).

## Results

**Fig. 1:**
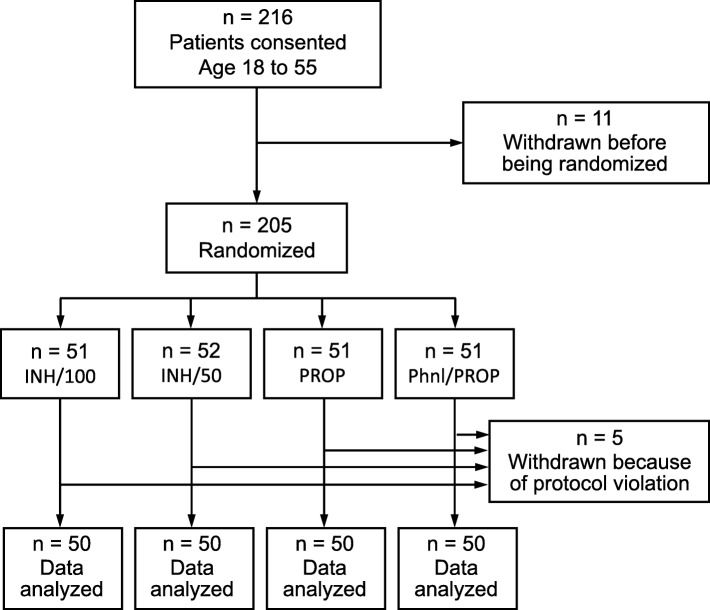
Consort Diagram. Eleven patients consented but were never randomized and not studied: 9: The first author was not available to perform the induction. 1: The case changed from a general anesthetic to a sedation case. 1: The surgeon did not want that patient to be in a clinical study. Five patients were induced and then withdrawn from analysis* because of protocol violations: 1: Airway difficulty during induction. 1: Additional propofol required; 2: Patients received more than 300 mL unwarmed IV fluid. 1: Forced air warming malfunction. *Four patients were withdrawn before T_15_ so that they had no post-induction temperature measurements. In one patient, there was only one temperature measurement at T_15_. This patient received more than 300 mL unwarmed IV fluid

After randomization and withdrawals, 50 patients in each group were analyzed (Fig. 1). Demographic and forced air warming data are presented in Table 1. The surgical procedures are presented in Table 5 Appendix 3.

**Table 1:** Demographics and forced air warming data of the 200 patients analyzed

Group	INH/100	INH/50	PROP	Phnl/PROP
Age (years)				
Mean (SD)	42.8 (10.1)	43.0 (8.6)	39.0 (11.2)	40.6 (9.1)
Range	22 to 55	26 to 55	18 to 55	20 to 55
Sex				
Male n(%)	20 (40)	31 (62)	20 (40)	21 (42)
ASA classification				
1 n (%)	1 (2)	3 (6)	10 (20)	2 (4)
2 n (%)	23 (46)	23 (46)	29 (58)	33 (66)
3 n (%)	16 (52)	24 (48)	11 (22)	15 (30)
BMI (kg/m^2^)				
Mean (SD)	31.9 (7.5)	31.2 (6.7)	26.8 (5.6)	29.9 (6.4)
Range	21.7 to 48.9	18.9 to 44.2	17.2 to 43.0	15.1 to 44.4
Preoperative screening temperature (°C)				
Mean (SD)	36.8 (0.3)	36.8 (0.3)	36.8 (0.3)	36.7 (0.3)
Use of upper body forced air warming (FAW) blanket (remaining patients used lower body FAW)				
n	32	39	29	30
(%)	(64)	(78)	(58)	(60)
Time from T_0_ until FAW turned on, (minutes)				
Mean (SD)	16.4 (7.0)	14.7 (7.0)	15.9 (7.9)	17.1 (7.7)
Range	5 to 45	4 to 45	4 to 44	6 to 40

**Fig. 2:**
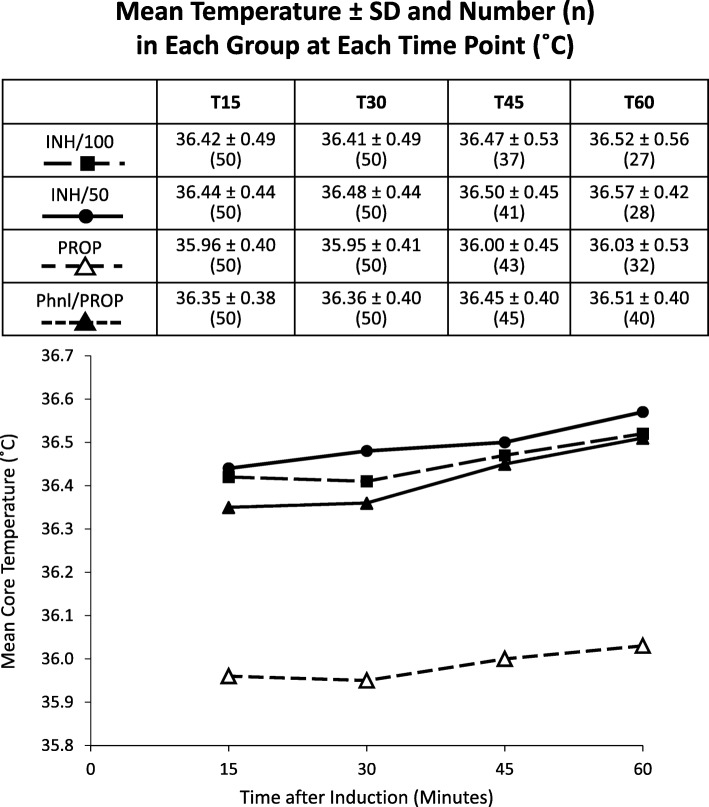
Mean Temperature ± SD and Number (n) in Each Group at Each Time Point (°C). In the three successive time intervals (T_15_ to T_30_, T_30_ to T_45_, and T_45_ to T_60_), the percentage of patients (all groups combined) whose temperature decreased were (37.5, 14.4, and 14.2% respectively). The percentage of patients whose temperature increased were (39.0, 55.4, and 59.1% respectively). The remaining patients had no temperature changes within these time intervals

Compared to group PROP, the three alternative induction groups each had higher mean core temperatures and fewer patients having at least one core temperature measurement < 36.0 °C in the first hour. At all four time points (T_15_, T_30_, T_45_, T_60_), the mean temperatures in group PROP were between 0.39 and 0.54 °C lower than in groups INH/100, INH/50 and Phnl/PROP (all *p* ≤ 0.0042 adjusted for multiple comparisons, Fig. 2, Table 2). There were no statistically significant differences in the mean temperatures between groups INH/100 and INH/50, INH/100 and Phyl/PROP, and INH/50 and Phyl/PROP at any time point (all *p* > 0.18). In group PROP, 60% of patients had at least one temperature < 36.0 °C in the first hour compared to 16% in each of groups INH/100, INH/50, and Phnl/PROP (all with an identical 44 percentage point difference, 95% CI 27% to 61%, *p* < 0.0001). In group PROP, 22% of patients had at least one temperature ≤ 35.5 °C, compared to 8% in group INH/100 (*p* = 0.09), 4% in INH/50 (*p* = 0.015), and 2% in Phnl/PROP (*p* = 0.004).

**Table 2:** Differences between the mean core temperature (°C) of each of three alternative induction groups and the standard propofol alone group at each time point^*^

Comparison groups	T_15_	T_30_	T_45_	T_60_
INH/100 minus PROP				
Difference (°C)	0.46	0.46	0.47	0.49
95% CI of difference	0.28 to 0.64	0.28 to 0.64	0.25 to 0.69	0.20 to 0.77
INH/50 minus PROP				
Difference (°C)	0.47^**^	0.52^**^	0.50	0.54
95% CI of difference	0.31 to 0.64	0.36 to 0.69	0.31 to 0.69	0.28 to 0.79
Phnl/PROP minus PROP				
Difference (°C)	0.39	0.41	0.45	0.47^**^
95% CI of difference	0.24 to 0.54	0.25 to 0.57	0.27 to 0.63	0.25 to 0.70

^*^Applying Bonferroni’s correction for the 12 multiple comparisons (3 groups compared to propofol only group at 4 time points) results in α = 0.05/12 = 0.0042. All 12 comparisons achieved statistical significance as *p* ≤ 0.001 < α = 0.0042 for each of the above comparisons

^**^These differences are correct to 2 decimal places. Because of rounding to two decimal places in Fig. 2, they differ by 0.01 from those that would be calculated using Fig. 2

No patient in any of these 4 groups had a core temperature > 37.5 °C at any time point. Apnea did not occur in either group INH/100 or INH/50 (0%, 95% CI 0% to 7.1% for each group).

Only blood pressures at T_1_ (and T_2_ if prior to airway intervention) were considered. In the first 2 min, treatment of hypotension (systolic BP < 85 mmHg) was required in 2 patients in Group PROP (4%, 95% CI 0.5% to 13.7%) and 1 patient in group Phnl/PROP (2%, 95% CI 0.05% to 10.6%). In group Phnl/PROP, only 1 patient’s blood pressure increased to a value > 180 mmHg and no patient suffered a reflex bradycardia ≤40 beats per minute. No patients in groups INH/100 or INH/50 (0%, 95% CI 0% to 7.1% for each group) required treatment for hypotension.

## Discussion

This effectiveness study found that in patients aged 18 to 55 years, inhalation inductions with sevoflurane or the administration of 160 mcg phenylephrine immediately prior to 2.2 mg/kg propofol each caused less redistribution hypothermia than intravenous inductions with propofol alone.

This study’s results are consistent with previous work [3, 4] and thus provide support for this study’s conclusion. Ikeda found a 0.7 °C average thermal advantage of sevoflurane inhalation inductions over intravenous propofol [3]. We found a slightly smaller (0.4 °C to 0.5°) advantage. That may reflect the use of forced air warming whereas Ikeda did not use FAW [3]. Also, Ikeda used a larger dose of propofol, which might have caused more vasodilation and thus more redistribution hypothermia. Sun found 64% of 58,814 patients had a temperature < 36 °C after 45 min, close to the 60% in group PROP; 29% were < 35.5 °C, close to the 22% in group PROP [4]. The small differences in results in these studies may in part reflect Sun’s patients having a higher mean age than study group PROP and/or random variation. Older patients have an increased risk for hypothermia [9, 14, 15].

Without patient warming, temperature decreases can continue for 3 h [16]. With the prompt initiation of forced air warming, we found most of the redistribution hypothermia occurred in the first 15 min. Within each group, the differences in mean core temperature between T_15_ and T_30_, T_30_ and T_45_, and T_45_ and T_60_ were small and clinically insignificant (Fig. 2).

We found a bolus dose of phenylephrine reduced redistribution hypothermia. Ikeda et al. found intraoperative phenylephrine infusion decreased the magnitude of redistribution hypothermia [17]. Ikeda concluded that even a short period of vasodilation can result in redistribution hypothermia [3]. The phenylephrine bolus opposed enough of the propofol induced vasodilation to reduce the amount of redistribution hypothermia. We administered a prior bolus dose of phenylephrine (about 10 s before propofol) without an infusion. Whether phenylephrine would be as effective if given after the propofol is not known. First, some vasodilation and heat transfer might have already occurred, and second, it is unknown if there is the same resultant vasodilation when phenylephrine is given after propofol.

Techniques that can reduce redistribution hypothermia now include prewarming [18–22], ketamine [23], etomidate [24], phenylephrine infusions [17], amino acid infusions [25], fructose [26], inhalation inductions [3], and bolus phenylephrine prior to propofol. None of these techniques solve the hypothermia problem fully. Combinations of these techniques may result in additional thermal benefit but have not been studied.

Inhalation inductions were performed gradually (i.e., without a primed circuit) for two reasons. First, apnea is unlikely to occur. Apnea never occurred in the 100 inhalation patients. Second, gradually increasing anesthetic depth likely contributes to hemodynamic stability, a potential benefit of inhalation inductions. Thwaites concluded that inhalation inductions were more hemodynamically stable than IV propofol inductions [27]. Retrospective studies found that adverse outcomes were associated with even short periods of hypotension, but not hypertension [28, 29]. Maheshwari et al. recently found that a substantial fraction of all hypotension occurred before surgical incision as a result of anesthetic management; this hypotension was associated with postoperative kidney injury [30]. We observed no hypotension (systolic BP < 85 mmHg) in any inhalation induction patient. Hypotension can occur rapidly with intravenous propofol inductions. Decreases in blood pressure with inhalation inductions are usually more gradual. Such gradual decreases could be addressed earlier, or prophylactically, before there is clinically important hypotension.

We found 160 mcg phenylephrine to be an effective dose in most Phnl/PROP patients. Small percentages of Phnl/PROP patients had a post-induction systolic blood pressure either < 85 mmHg or > 180 mmHg. An optimal phenylephrine dose (e.g., weight based) would minimize hypotensive and hypertensive events and still maintain the thermal benefit. We studied only one dose of phenylephrine.

In the multivariable analysis, neither BMI nor sex was associated with the degree of redistribution hypothermia. This indicates that differences in BMI and sex between treatment groups were not responsible for the differences in mean core temperatures (redistribution hypothermia) between groups. We found patients were susceptible to redistribution hypothermia regardless of BMI. Because it takes more heat transfer to change the temperature of a heavier patient, it is commonly believed that obese patients are more resistant to temperature change. However, a different process is dominant during the initial redistribution hypothermia phase. Many obese patients have substantial muscle mass in their periphery to move their heavy body parts. The relatively little blood flow in adipose tissue may prevent meaningful temperature buffering during the redistribution hypothermia phase.

## Limitations

We studied hypothermia during surgery, not surgical outcome. Since hypothermia causes adverse outcomes [1, 2], it is plausible that the studied alternative induction techniques will result in superior clinical outcomes than propofol by keeping patients warmer. It remains to conduct randomized controlled trials addressing all major aspects of anesthesia care (e.g., hemodynamics, post-operative nausea and vomiting) and whether clinical outcomes improve using any of the alternative induction techniques.

Although our results suggest a possible hemodynamic benefit of these alternative induction techniques, many more patients need to be studied to demonstrate that benefit.

In randomization, allocation of treatment to individuals is left purely to chance and not systematically biased by deliberate selection of patients. Despite randomization, there were imbalances in sex and BMI. These imbalances may have been due either to chance or to insufficient group size. Increasing the size of the study reduces the probability of baseline differences in both measured and unmeasured variables, but cannot assure balanced groups. Baseline differences are only important if they affect the study conclusions. In this study, these imbalances did not provide an alternative explanation to the three treatments’ thermal advantage over propofol. In bivariate analyses, neither sex nor BMI were associated with the observed temperature differences. Their inclusion in the multivariable analysis did not affect the temperature differences between propofol and the other treatment groups, Thus, despite baseline differences, neither sex nor BMI provides an alternative explanation. Although there may be unmeasured confounding, the large and consistent differences between propofol and the alternative treatment groups makes it unlikely that the study conclusions will change.

Although the study design reduced the risk of selection bias, the possibility of measurement bias still exists. Having one caregiver (the first author) performing the randomization, providing anesthetic care, and recording outcomes is a potential source of bias. However, since the next study patient was already in the operating room at the time of random assignment, the caregiver could not affect the random allocation. By having a single caregiver, the manner of induction and all the tasks performed at the beginning of the case were more uniform than if the tasks were performed by multiple caregivers. The temperatures recorded were objective and not affected by who recorded the data.

We did not control for “depth of anesthesia”. A greater depth of anesthesia likely results in more vasodilation and thus more redistribution hypothermia (and hypotension). If inductions achieve only the minimum necessary depth, it is plausible there may be less redistribution hypothermia (and hypotension). In this effectiveness study, we titrated the maintenance dose of anesthetic by vital signs, as is common in clinical practice. Titrating anesthetic doses to anesthetic depth may yield different results. A given depth of anesthesia can be achieved by varying the type and dose of anesthesia. We studied only one dose of propofol. It is plausible that using a lower dose of propofol and/or sevoflurane for induction will result in less redistribution hypothermia. Kazama et al. demonstrated that anesthetic inductions can be accomplished with a smaller dose of propofol than used in this study [31].

There were a variety of different surgeries, but no major intraabdominal or intrathoracic surgeries (with their greater potential for intraoperative heat loss). Since patients with different surgeries were treated similarly and had comparable thermal exposure in the first 15 min post induction, the impact of the specific surgery should be minor.

We did not study intrathoracic or major intraabdominal surgeries where there is greater thermal stress. We can make no statement as to whether the initial thermal benefit is well maintained in such cases.

We did not measure pre-induction (baseline) core temperatures. Although studies similar to this one often compare the changes from baseline temperature, we did not. We measured the core temperatures at 4 times after induction. To address the lack of pre-test core temperature measurements, we used a post-test only randomized trial. Since the randomization makes it likely that the baseline temperatures are similar in the four groups, the observed post-induction differences are probably due to the different induction techniques. Our experimental design, while less common, is a standard design that “contains no threats to internal validity” [11].

We performed “as treated” analyses on 200 of the 205 (97.6%) randomized patients. Those 5 excluded patients provided no valid temperature data. Four patients had no post-induction temperature measurements because they were withdrawn before T_15_. In one patient, there was only one temperature measurement at T_15_. This patient received more than 300 mL unwarmed IV fluid. This makes it very unlikely that the results differ substantially from an intention-to-treat analysis. With the exclusion of these five patients, the 200 patients presented in Table 1 are exactly those we analyzed. Thus, the randomization combined with the bivariate and multivariable statistical results support the study conclusion that the three alternative inductions produced higher intraoperative temperatures than propofol alone.

## Conclusions

This study makes evident the thermal benefits of inhalation inductions and prophylactic bolus phenylephrine administration over standard intravenous propofol alone inductions in adults age 18 to 55 inclusive. This offers quick, simple, and easy to use partial solutions to the on-going problem of intraoperative hypothermia.

## Data Availability

The datasets generated and analyzed during the current study are available in the Mendeley repository: https://data.mendeley.com/datasets Search “Redistribution hypothermia”. Abstract uploaded to: http://www.persistent-identifier.nl/?identifier=urn%3Anbn%3Anl%3Aui%3A13-lo-lchp

## Appendix 1

**Table 3:** Study groups

Group	Induction technique
INH/100	Inhalation: 8% sevoflurane in 100% oxygen
INH/50	Inhalation: 8% sevoflurane in 50% oxygen and 50% nitrous oxide
PROP	Intravenous: 2.2 mg propofol
Phyl/PROP	Intravenous: 2.2 mg propofol preceded by 160 mcg phenylephrine

## Appendix 2

**Table 4:** Inclusion and exclusion criteria

Inclusion criteria	
Age 18 to 55 years inclusive	
Scheduled for general anesthesia where 50% nitrous oxide in oxygen would be used	
Endotracheal intubation or laryngeal mask airway insertion would be used	
Afebrile (preoperative oral or temporal scan temperature between 36.2 and 37.4 °C inclusive)	
Positioned supine or lithotomy	
Forced air warming would be used	
Expected duration of anesthetic to be at least 60 min	
Exclusion criteria	
Emergency surgery or any other aspiration risk	
Age < 18 years or > 55 years	
Pregnant	
Incarceration	
Febrile illness	
Anticipated difficult airway	
Contraindication to nitrous oxide use	
Contraindication to nasal instrumentation	
Nasal surgery	
Current or recent epistaxis	
Requirement for foreign language interpreter	
Allergy to propofol	
Malignant hyperthermia risk	
Inability to oxygenate on less than 50% oxygen	
Cardiac surgery	
Neuro-surgery	
Receiving vasoactive infusions	
Significant valvular heart disease	
Unstable cardiac disease	
Requiring prone or lateral positioning	
Inability to provide informed consent	
Inability to use forced air warming	
Untreated hypo- or hyper-thyroidism	
ASA class 4, 5 or 6^a^	
Anticipated inability to tolerate any of the 4 different anesthetic induction options in this study	

^a^Patients with end stage renal disease on dialysis were classified as ASA 3

## Appendix 3

**Table 5:** List of surgeries

Procedure	Groups having at least one patient having the listed procedure are denoted by an x			
	INH/100	INH/50	PROP	Phnl/PROP
UROLOGIC				
Cystoscopic surgery	x	x	x	x
Penile procedures	x	x	x	
Suprapubic tube placement	x			
Scrotal procedures	x			
Urethroplasty		x	x	x
ORTHOPEDIC				
Lower extremity orthopedics	x	x	x	x
Upper extremity orthopedics	x	x	x	x
Anterior cervical discectomy and fusion				x
GYNECOLOGIC				
Vulvoplasty or excision of lesion	x	x		
Dilation and curettage, hysteroscopy	x	x	x	x
Loop endocervical excision procedure	x	x		
Endocervical curettage	x	x		
Hysterectomy	x	x		x
Myomectomy		x	x	x
VASCULAR				
Dialysis access related procedures	x	x	x	x
Lower extremity vascular – open procedures		x		
Radiofrequency ablation and/or Lower extremity phlebectomies	x	x	x	
Lower extremity amputations	x	x		
DENTAL/ENT	x	x	x	
THORACIC				
Endobronchial ultrasound	x	x	x	
GENERAL SURGERY				
Vacuum assisted closure change	x			
Non-cavitary procedures	x	x		x
Inguinal hernia	x			x
Breast	x	x	x	x

## Abbreviations

BMI: Body mass index
BP: Blood pressure
C: Centigrade
CI: Confidence interval
FAW: Forced air warming
Hg: Mercury
INH/100: Study group induced with 100% oxygen
INH/50: Study group induced with 50% oxygen and 50% nitrous oxide
IV: Intravenous
kg: Kilogram
LMA: Laryngeal mask airway
LPM: Liters per minute
m: Meter
mcg: Microgram
mg: Milligram
mL: Milliliter
mm: Millimeter
N_2_O: Nitrous oxide
O_2_: Oxygen
Phnl/PROP: Study group induced with 160 mcg phenylephrine followed by 2.2 mg/kg IV propofol
PROP: Study group induced with 2.2 mg/kg IV propofol
SCIP: Surgical Care Improvement Project
SD: Standard deviation
T_x_: Time x minutes after the start of anesthetic induction
