# Correction to: Induction techniques that reduce redistribution hypothermia: a prospective, randomized, controlled, single blind effectiveness study

**DOI:** 10.1186/s12871-021-01327-4

**Published:** 2021-04-12

**Authors:** Jonathan V. Roth, Leonard E. Braitman, Lacy H. Hunt

**Affiliations:** 1 Department of Anesthesiology, Albert Einstein Medical Center, 5501 Old York Road, Philadelphia, PA USA; 2 Albert Einstein Healthcare Network, Philadelphia, PA USA; 3 Sidney Kimmel Medical School of Thomas Jefferson University, Philadelphia, PA USA; 4 Office for Research and Technology Development, Albert Einstein Medical Center, 5501 Old York Road, Philadelphia, PA USA

**Correction to: BMC Anesthesiol (2019) 19:203**

**https://doi.org/10.1186/s12871-019-0866-8**

Following publication of the original article [1], the authors reported errors.

In Table 3 in Appendix 1, the dose of propofol for the intravenous inductions should be “2.2 mg/kg”, not “2.2 mg”.

In our study, we compared three alternative induction techniques (inhalation induction with 8% sevoflurane in 100% oxygen [Group INH/100], inhalation induction with 8% sevoflurane in 50% oxygen and 50% nitrous oxide [Group INH/50], and 2.2 mg/kg intravenous propofol that was preceded by 160 mcg phenylephrine [Group Phnl/PROP]) to the standard 2.2 mg/kg propofol alone induction [Group PROP] and demonstrated that the three alternative induction techniques reduced redistribution hypothermia by an average of 0.4 to 0.5°C in the first hour of anesthesia [1]. The incidence of any patient having at least one core temperature reading <36.0°C in the first hour was 60% in Group PROP and 16% in each of the three alternative groups. These four groups of 50 patients each were aged 18 to 55 years.

At the same time, as an exploratory endeavor we studied two groups of 50 patients each aged >55 years who received an inhalation induction with either 8% sevoflurane in 100% oxygen [Group INH/100>55] or 8% sevoflurane in 50% oxygen and 50% nitrous oxide [Group INH/50>55]. Because the dose of propofol would often need to be reduced in patients age >55 years, we did not study anesthetic inductions with propofol. Because some older patients would need different propofol doses, no single dose propofol group can be constructed. Therefore, it would not be possible to perform a randomized comparison that compares a fixed dose of propofol to an inhalation induction. Thus, it was not possible to address the primary study question (comparing alternative induction techniques to standard propofol alone) because there was no propofol only group in patients > 55 years.

The age 18 to 55 years subjects and the age >55 years subjects were planned and recruited according to the trial design described in the trial registration. Although we reported all the results to clinical-trials.gov (NCT02331108) and presented our results that included the age >55 years groups in abstracts [2, 3], we did not include the age >55 years results in our article^1^ because they could not be used to address the primary research question. However, for consideration of the transparency of the trial and since we do consider the age >55 years results useful, these results should be published. The two main results for the age >55 years groups are:

1. Figure 2 now includes groups INH/50>55 and INH/100>55.
2. In both age >55 years groups, 28% of patients ever had at least 1 core temperature measurement <36.0°C in the first hour.

**Fig. 2:**
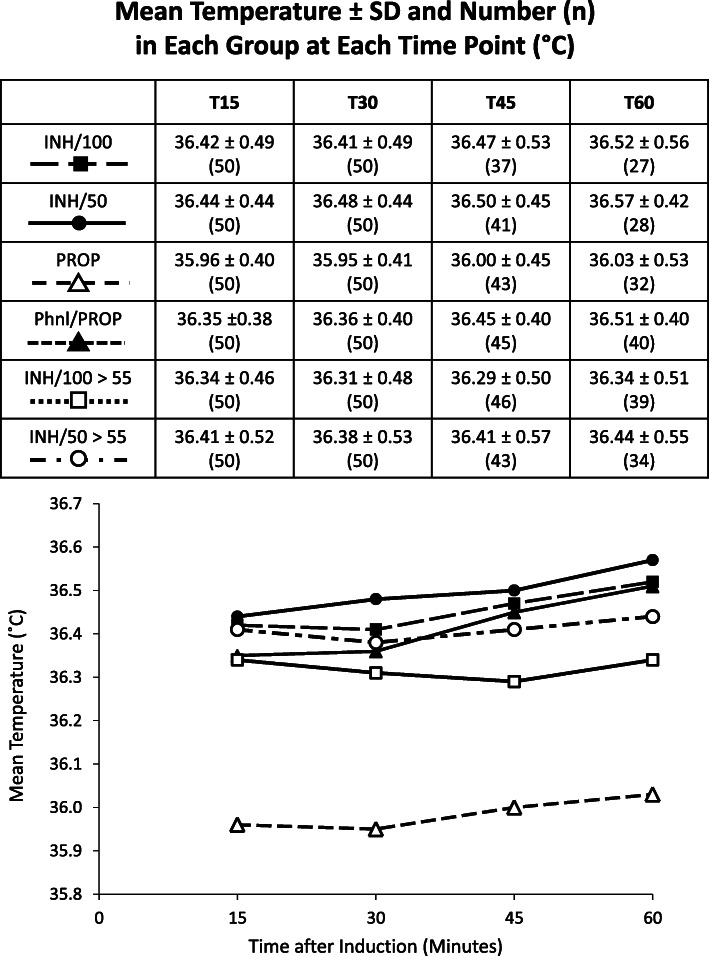
T15, T30, T45, and T60 designate 15, 30, 45, and 60 minutes after the initiation of anesthetic induction. Group INH/100 are subjects age 18 to 55 years who had an inhalation induction with 8% sevoflurane in 100% oxygen. Group INH/50 are subjects age 18 to 55 years who had an inhalation induction with 8% sevoflurane in 50% oxygen and 50% nitrous oxide. Group PROP are subjects age 18 to 55 years who had an intravenous induction with 2.2 mg/kg propofol. Group Phnl/PROP are subjects age 18 to 55 years who had an intravenous induction with 2.2 mg/kg propofol that was preceded by 160 mcg phenylephrine. Group INH/100>55 are subjects age >55 years who had an inhalation induction with 8% sevoflurane in 100% oxygen. Group INH/50>55 are subjects age >55 years who had an inhalation induction with 8% sevoflurane in 50% oxygen and 50% nitrous oxide

Numerically, compared to the age 18 to 55 years inhalation subjects, the average mean core temperatures were lower in the age > 55 years inhalation subjects (Figure 2), and the older subjects also had a greater percentage of having at least 1 core temperature measurement <36.0°C in the first hour (28% vs 16%). This is consistent with the belief that older patients are more prone to hypothermia than younger patients. Even so, compared to the 18 to 55 years old propofol alone subjects, numerically the older inhalation subjects stayed warmer (Figure 2) and had a lower percentage of having at least 1 core temperature measurement <36.0°C in the first hour (28% vs 60%). This suggests inhalation inductions have efficacy in reducing redistribution hypothermia in patients >55 years old.

The age 18 to 55 years and age >55 years subjects were randomized separately from different populations. The study of the older age subjects has absolutely no impact on what we published.^1^ It did not affect the recruitment, randomization, conduct, or analysis of the study of the 18 to 55 years old. The age >55 years results do not affect the main conclusion of the published article.
